# Chemical composition of barley and co-products from barley, corn, and wheat produced in South-East Asia or Australia

**DOI:** 10.5713/ab.23.0201

**Published:** 2023-08-24

**Authors:** Natalia S. Fanelli, Leidy J. Torres-Mendoza, Jerubella J. Abelilla, Hans H. Stein

**Affiliations:** 1 Department of Animal Sciences, University of Illinois, Urbana IL 61801, USA; 2 DSM Nutritional Products, Mapletree Business City 117440, Singapore

**Keywords:** Barley, Barley Co-products, Chemical Composition, Corn Co-products, Wheat Co-products

## Abstract

**Objective:**

A study was conducted to determine the chemical composition of barley and co-products from barley, corn, and wheat produced in South-East Asia or Australia, and to test the hypothesis that production area or production methods can impact the chemical composition of wheat co-products.

**Methods:**

Samples included seven barley grains, two malt barley rootlets, one corn gluten feed, one corn gluten meal, one corn bran, eight wheat brans, one wheat mill mix, and four wheat pollards. All samples were analyzed for dry matter, gross energy, nitrogen, amino acids (AA), acid hydrolyzed ether extract, ash, minerals, starch, and insoluble dietary fiber and soluble dietary fiber. Malt barley rootlets and wheat co-products were also analyzed for sugars.

**Results:**

Chemical composition of barley, malt barley rootlets, and corn co-products were in general similar across countries. Wheat pollard had greater (p<0.05) concentrations of tryptophan, magnesium, and potassium compared with wheat bran, whereas wheat bran had greater (p<0.05) concentration of copper than wheat pollard. There were no differences in chemical composition between wheat bran produced in Australia and wheat bran produced in Thailand.

**Conclusion:**

Intact barley contains more starch, but fewer AA, than grain co-products. There were only few differences in the composition of wheat bran and wheat pollard, indicating that the two ingredients are similar, but with different names. However, corn gluten meal contains more protein and less fiber than corn bran.

## INTRODUCTION

Demand for feed grain for livestock has increased in Australia and animal feed accounts for the consumption of two-thirds of all domestic Australian crop production [1]. Feeding is also one of the most challenging aspects of livestock farming in Asia, which is dependent on the availability of feed ingredients [2]. Animal production in this region is primarily focused on crop-animal systems, which provides a variety of grain co-products that can be used as feed ingredients in animal diets [2]. Processing of grain and grain co-products involves harvesting, cleaning, milling or grinding, and separating the different components to obtain desired end products such as flour, bran, and germ, but the left-over co-products are often used as feed for livestock [2].

Barley is a major cereal grain mostly produced in the European Union, Russia, Canada, and Australia [3]. It is used in animal diets and for human consumption, as well as for distilling and brewing. Nutrient composition of barley depends on factors including variety, environment, and yield [4]. Malt barley rootlets, a co-product obtained at the final stage of the barley malting process, consist of the dried shoots and rootlets of the sprouted grain [5], and can provide protein in diets for livestock. Co-products from the corn milling industry such as corn bran, corn gluten meal, and corn gluten feed are also available [6]. Corn bran is the pericarp-enriched fraction derived from the dry- or wet-milling industry, whereas corn gluten feed and corn gluten meal are co-products from the wet-milling industry [6]. Corn gluten feed is the remaining part of the corn grain after extraction of most of the starch, gluten, and germ, whereas corn gluten meal is the dried residue that is left after manufacture of starch and germ [7].

Wheat is also an important agricultural crop in some Australian and South-East Asian regions [8]. Wheat co-products from dry milling of wheat include wheat bran, which is the primary pericarp layer of wheat grain [8], and wheat pollard (hard or soft) known as fine bran [9]. Mill mix, also known as mill run, is another wheat co-product from the flour milling process that is a combination of bran and shorts fractions [10].

Despite the importance of grain and grain co-products in the nutrition of livestock and poultry, there is limited information about the chemical composition of grain and grain co-products produced in South-East Asia and Australia. In addition, most studies regarding chemical composition have focused only on a few ingredients from specific locations. Likewise, in the analysis of feed ingredients, components usually do not add up to 100% because not all nutrients are analyzed. Therefore, determining the complete chemical composition of feed ingredients from South-East Asia or Australia is important for livestock nutrition because this will aid in formulation of balanced diets that meet the nutritional needs of the animals. Therefore, the objectives of this study were to determine the chemical composition of barley and co-products from barley, corn, and wheat from South-East Asia or Australia, and to test the hypothesis that there are differences among grain co-products produced in different countries.

## MATERIALS AND METHODS

### Description of samples

Barley and co-products from barley, corn, and wheat (between 100 and 300 grams of each ingredient) were collected from suppliers in South-East Asia or Australia and delivered to DSM Nutritional Products, Singapore. Samples were labelled and then shipped to the University of Illinois, Urbana, IL, USA, where most of the chemical analyses were conducted. Samples included seven sources of barley from Australia and Indonesia, two sources of malt barley rootlets from Australia and Indonesia, one sample of corn gluten feed from Indonesia, one sample of corn gluten meal and a sample of corn bran from the Philippines, eight sources of wheat bran from Australia, Indonesia, the Philippines, Thailand, and Vietnam, one sample of wheat mill mix from Australia, and four sources of wheat pollards from the Philippines.

### Chemical analysis

Samples of all ingredients were finely ground and analyzed for dry matter (Method 930.15) and ash (Method 942.05) [11]. Gross energy was analyzed using an isoperibol bomb calorimeter (Model 6400, Parr Instruments, Moline, IL, USA). Samples were analyzed for amino acids (AA) [Method 982.30 E (a, b, c)] [11] on a Hitachi AA Analyzer (Model L8800; Hitachi High Technologies America Inc., Pleasanton, CA, USA) and nitrogen was analyzed by combustion (Method 990.03) [11] using a LECO FP628 Nitrogen Analyzer (LECO Corp., Saint Joseph, MI, USA). Methionine and Cystein were determined as methionine sulfone and cysteic acid after cold performic acid oxidation overnight before hydrolysis [11]. Crude protein was calculated as nitrogen×6.25. The acid hydrolyzed ether extract (AEE) was analyzed using 3*N* HCl (Ankom^HCl^; Ankom Technology, Macedon, NY, USA) followed by crude fat extraction using petroleum ether (Ankom^XT15^; Ankom Technology, USA). Insoluble dietary fiber and soluble dietary fiber were quantified according to method 991.43 [11] using the Ankom^TDF^ Dietary Fiber Analyzer (Ankom Technology, USA). Total dietary fiber was calculated as the sum of insoluble and soluble dietary fiber. Minerals were analyzed (Method 985.01 a, b, and c) [11] using inductively coupled plasma-optical emission spectrometry (ICP-OES; Avio 200, PerkinElmer, Waltham, MA, USA). Sample preparation included dry ashing at 600°C for 4 h (Method 942.05; 10) [11] and wet digestion with nitric acids (Method 3050 B) [12]. Total starch was analyzed using the glucoamylase procedure (Method 979.10) [11]. Sugars including glucose, fructose, maltose, sucrose, stachyose, and raffinose were analyzed in malt barley rootlets and wheat co-products using high-performance liquid chromatography (Dionex App Notes 21 and 92).

### Calculations and statistical analysis

For each feed ingredient, analyzed proximate components were added and subtracted from the concentration of dry matter to calculate the rest fraction according to the following equations:


$$\begin{array}{l} \text{Rest }{\text{fraction}}_{\text{barley and corn co-products}}\\ =[\text{Dry matter}-(\text{crude protein}+\text{AEE}+\text{ash}\\ +\text{total dietary fiber}+\text{total starch})]. \end{array}$$

$$\begin{array}{l} \text{Rest }{\text{fraction}}_{\text{malt barley rootlets and wheat co-products}}\\ =[\text{Dry matter}-(\text{crude protein}+\text{AEE}+\text{ash}\\ +\text{total dietary fiber}+\text{total starch}+\text{glucose}\\ +\text{fructose}+\text{maltose}+\text{sucrose}+\text{stachyose}+\text{raffinose})]. \end{array}$$

The rest fraction of malt barley rootlets and wheat coproducts differs from rest fraction of barley and corn co-products because these ingredients do not contain a considerable amount of free sugars. To allow for statistical comparison, all samples were adjusted to 90% dry matter because this is a typical value for grains and allows for a direct comparison without the influence of moisture. If two or more samples of the same ingredient from one country were available, the coefficient of variation and the average were calculated.

Normality of residuals was verified using the UNIVARIATE procedure (SAS 9.4 Institute Inc., Cary, NC, USA). Data were analyzed by analysis of variance using the F-test in the PROC MIXED procedure in SAS to test differences among wheat co-products and differences in composition of wheat co-products from different countries. The replicate sample was the experimental unit for all analyses. The feed ingredient or country was the fixed effect, and the replicate sample was the random effect. Means were calculated using the LSMEANS statement in SAS. Results were considered significant at p<0.05.

## RESULTS

The nutrient composition of barley grain from Australia and Indonesia was not different, with the exception that the barley grain from Australia had a greater concentration of total starch and a lower concentration of total dietary fiber than the barley grain from Indonesia (Table 1). The coefficient of variation for the analyzed components of barley grain from Australia was low, except for ash and most minerals, accounting for less than 30%. The average rest fraction for the barley grain samples was less than 2%.

The main nutrients in malt barley rootlet samples were crude protein (25% to 26%) and total dietary fiber (36% to 38%), the majority of which was insoluble dietary fiber (Table 2). Starch, AEE, sugars, and minerals were also present, but at lower concentrations (1% to 10%). The average rest fraction in these samples was very low, accounting for less than 1%.

The main nutrients in corn gluten feed were crude protein and insoluble dietary fiber (Table 3). The most abundant nutrient in corn gluten meal was also crude protein, but total dietary fiber in corn gluten meal was low, whereas the main nutrients in corn bran were insoluble dietary fiber and starch. Minerals and AEE were also present in all corn co-products, but at lower concentrations than other nutrients. All corn co-products had a rest fraction that was close to or less than 3%.

The coefficient of variation for the analyzed wheat bran from Australia was low, accounting for less than 30%, with the exception of starch, soluble dietary fiber, and all sugars, which varied more among samples (Table 4). However, wheat bran from Thailand had on average a coefficient of variation (of around 40%) for most nutrients. The coefficient of variation among wheat pollard samples from the Philippines was low, except for starch, soluble dietary fiber, and some sugars and minerals, accounting for less than 30% (Table 5). The average rest fraction in wheat bran and wheat pollard was very low, accounting for less than 0.5%, regardless of origin.

No differences were observed between wheat bran samples from Australia and Thailand (Table 6). Wheat pollard had greater (p<0.05) concentrations of tryptophan, magnesium, and potassium when compared with wheat bran, but wheat bran had greater (p<0.05) concentration of copper than wheat pollard.

## DISCUSSION

The chemical composition of barley and malt barley rootlets was within the range of published values [3,4,13–17]. However, the total dietary fiber in barley used in this study was greater than that reported by McGhee and Stein [13] and NRC [14]. The concentration of starch is usually greater in barley grown in temperate climates than in subtropical or tropical climates, whereas the opposite is the case for dietary fiber, which may be the reason for the greater fiber analyzed in the present samples [15].

The chemical composition of corn co-products was also within the range of published data [14,16,18,19], although corn gluten feed had lower total starch and greater total dietary fiber concentrations, and corn gluten meal had lower total starch and greater AEE than observed in some published studies. Likewise, the chemical composition of wheat co-products was within the range of published values [8,10,14, 16,20–23], but the starch content of wheat co-products in this study was lower than reported by Rostagno et al [16]. The chemical components of all analyzed ingredients were close to 100%, indicating that most nutrients were accounted for because the sum of all analyzed nutrients were close to the dry matter content [24].

### Barley and malt barley rootlets

Barley is a major grain that is used for human consumption, livestock feed, and brewing [4]. Australia is one of the world’s largest barley producers and the country is currently the largest exporter of barley. In 2022, the total barley export from Australia was 8.5 million metric tons [25]. Barley, is therefore, an important crop in Australia. Many factors influence the chemical composition of barley, including variety, yield, and environment. The observation that the barley grain from Indonesia had greater crude protein and total dietary fiber, but contained less starch than barley from Australia, indicates that barley from Indonesia had lower yields, which generally increases protein and fiber levels while decreasing starch content [4]. Barley has a high concentration of starch and protein is close to that in wheat and greater than corn, but because of the high fiber in barley, feeding barley to animals sometimes results in reduced dietary energy intake [26]. However, energy digestibility in pigs can be increased if particle size is reduced [27].

The malt barley rootlets, also known as malt culms, represent an important fraction of alternative ingredients for animal diets [5], and they are mostly used for ruminants. However, barley rootlets may also be used at low levels for finishing pigs, but low AA digestibility and poor palatability prevent greater inclusion [3]. The protein and total dietary fiber concentrations of malt barley rootlets used in this study averaged 25.9% and 37.5%, respectively. The high concentration of sugars in barley rootlets is due to the malting process that converts starch into soluble sugars [4].

### Corn co-products

Corn demand has increased as a result of bioethanol production, as well as increased use in the food and animal feed industries [28]. Based on USDA-FAS [29], the Philippines is recognized as a significant harvesting region in South-East Asia, with a corn production of approximately 8.1 million metric tons in 2022. The corn bran used in this study had an oil content of 9.9%, which is within the typical range, with values ranging from 8% to 12% [14]. However, corn bran has lower digestible energy and AA compared with corn when fed to growing pigs because of greater dietary fiber [30].

The high protein and low total dietary fiber in corn gluten meal were in agreement with published data [19]. Protein digestibility is high in corn gluten meal [6,14], but protein quality is poor, with tryptophan and lysine being limiting AA. Corn gluten meal is mostly used as an alternative to other plant or animal-based proteins in diets for ruminants, but not often in diets for pigs and poultry due to the poor protein quality [18]. The corn gluten feed usually has a high concentration of total dietary fiber and protein can range between 17% and 24% depending on the milling process and relative proportions of bran, steep liquor, and other components included in the final product [7]. Corn gluten feed is an important ingredient in ruminant diets, but it has a lower nutritional value for monogastrics because of poor AA balance and low energy due to the high concentration of fiber [6,19].

### Wheat co-products

Wheat milling generates co-products that are needed in the food industry, in animal feed, in bioethanol production, or in other industries [4]. Australia accounts for 13% of global wheat exports, but as a result of the current conflict between Ukraine and Russia, as well as port closures, wheat prices have increased, with importers reducing purchases and relying on existing stocks. This is currently the case in most South-East Asian countries where wheat is not a major crop [25]. Wheat composition is affected by variety, seasonal growing conditions, yield, fertilizer use, and sample cleanliness. Wheat bran and pollard are the two wheat co-products that are most used as livestock feed and can provide energy as well as some of the indispensable digestible AA in pig and poultry diets [21].

The observation that there were no differences in chemical composition between wheat bran from Australia and Thailand, indicates that the processing method used in the two countries is similar. Wheat bran is a fibrous co-product, containing the husk and some adhering endosperm [7], and it is generally palatable, has a moderate protein content, and its fiber content is excellent for digestive problems in monogastric animals including horses, but the high concentration of insoluble dietary fiber results in low energy digestibility [20,21,23] and reduced concentration of digestible energy for pigs [31].

Wheat pollard makes up the fraction of wheat that is not used in wheat flour production, and is the main co-product produced by flour milling. The composition of wheat pollard may depend on the type of flour produced and whether or not the germ is added. Wheat co-products are known by different names, including pollards, sharps, middlings, and shorts and its feeding value is determined by its fiber and starch contents [4]. Because wheat pollard is assumed to be less fibrous and with greater concentration of gluten than wheat bran, it was expected that the composition would be different. However, the observation that the wheat bran and wheat pollard that were analyzed in this study generally had the same nutrient composition indicates that the processing used to produce the two co-products was not different, or that suppliers are unaware of the differences and may mislabel them. Wheat co-products can be used for ruminant and pig diets, but high levels of inclusion can increase transit rate due to the high fiber content, resulting in decreased digestibility of nutrients [21].

## CONCLUSION

The chemical composition of barley and co-products from barley, corn, and wheat produced in South-East Asia or Australia was typically consistent with reported values for similar co-products from other regions of the world. There were no differences between wheat bran produced in Australia and Thailand. There were only a few differences between wheat bran and wheat pollard, indicating that the processing used to produce these two ingredients was not different or that there may be a lack of knowledge of origin when labeling products for sale. As a result, it is critical that chemical analysis be performed when using alternative ingredients to meet expectations and ensure feeding quality.

## Figures and Tables

**Table 1:** Analyzed nutrient composition of barley grain, as-fed basis^1)^

Item (%)	Australia								Indonesia

	Sample 1	Sample 2	Sample 3	Sample 4	Sample 5	Sample 6	CV	Average	
Dry matter	89.16	89.14	88.83	88.86	89.14	89.16	0.18	89.05	89.45
Gross energy (kcal/kg)	4,012	4,031	4,048	4,055	3,931	3,965	1.23	4,007	3,962
Crude protein	10.98	11.04	11.22	11.28	8.62	10.57	9.52	10.62	11.19
AEE	2.05	2.14	2.00	2.17	2.03	2.53	9.09	2.15	2.20
Ash	2.14	2.12	1.95	2.01	1.79	3.78	32.06	2.30	2.98
Carbohydrates									
Total starch	54.10	55.23	54.31	53.27	58.16	53.50	3.29	54.76	47.39
Insoluble dietary fiber	14.94	15.04	16.11	15.29	15.95	14.64	3.81	15.33	22.14
Soluble dietary fiber	3.13	3.94	3.75	3.44	2.83	2.83	14.13	3.32	2.62
Total dietary fiber	18.07	18.98	19.86	18.74	18.78	17.46	4.39	18.65	24.75
Rest fraction^2)^	2.64	0.44	0.70	2.58	0.59	2.11	-	1.51	1.44
Indispensable AA									
Arginine	0.52	0.51	0.52	0.52	0.40	0.58	11.57	0.51	0.54
Histidine	0.24	0.24	0.24	0.24	0.19	0.24	8.81	0.23	0.24
Isoleucine	0.41	0.41	0.44	0.42	0.32	0.43	10.68	0.41	0.41
Leucine	0.75	0.75	0.79	0.76	0.60	0.77	9.31	0.74	0.72
Lysine	0.45	0.44	0.45	0.43	0.35	0.58	16.45	0.45	0.48
Methionine	0.19	0.17	0.18	0.17	0.14	0.21	13.23	0.18	0.18
Phenylalanine	0.57	0.58	0.62	0.59	0.42	0.53	12.84	0.55	0.55
Threonine	0.36	0.36	0.36	0.35	0.29	0.39	9.42	0.35	0.38
Tryptophan	0.10	0.10	0.10	0.10	0.08	0.09	8.81	0.10	0.09
Valine	0.56	0.55	0.56	0.55	0.43	0.53	9.47	0.53	0.54
Dispensable AA									
Alanine	0.44	0.43	0.44	0.43	0.35	0.45	8.67	0.42	0.48
Aspartic acid	0.68	0.67	0.65	0.63	0.53	0.81	13.67	0.66	0.73
Cysteine	0.24	0.24	0.25	0.23	0.19	0.23	9.12	0.23	0.22
Glutamic acid	2.54	2.58	2.81	2.66	1.88	2.16	14.29	2.44	2.35
Glycine	0.45	0.44	0.46	0.45	0.36	0.45	8.57	0.44	0.47
Proline	1.12	1.14	1.31	1.23	0.82	0.85	18.60	1.08	1.02
Serine	0.41	0.40	0.43	0.42	0.33	0.42	9.10	0.40	0.42
Tyrosine	0.26	0.26	0.27	0.28	0.19	0.27	12.83	0.26	0.27
Minerals									
Calcium	0.03	0.04	0.05	0.05	0.03	0.02	33.03	0.04	0.06
Phosphorus	0.38	0.33	0.29	0.26	0.26	0.30	15.13	0.30	0.38
Magnesium	0.15	0.11	0.09	0.10	0.10	0.12	19.14	0.11	0.12
Potassium	0.70	0.37	0.44	0.48	0.40	0.53	24.44	0.49	0.55
Sodium	0.02	0.01	0.03	0.03	0.04	0.01	51.90	0.02	0.02
Sulfur	0.08	0.01	0.01	0.02	0.02	0.05	88.01	0.03	0.02
Cooper (mg/kg)	8.28	7.19	6.22	6.35	4.40	5.66	20.80	6.35	6.24
Iron (mg/kg)	78.00	53.24	32.18	36.02	36.53	28.92	42.11	44.15	71.60
Manganese (mg/kg)	27.16	15.99	11.72	12.53	12.64	49.95	69.27	21.67	14.54
Zinc (mg/kg)	34.94	24.46	21.22	20.81	16.31	17.70	29.68	22.57	26.58

CV, coefficient of variation; AEE, acid hydrolyzed ether extract; AA, amino acids.

1) Except for dry matter, all values were adjusted to 90% dry matter.

2) Rest fraction = calculated using the following equation: [Dry matter – (crude protein + AEE + ash + total dietary fiber + total starch)].

**Table 2:** Analyzed nutrient composition of malt barley rootlets, as-fed basis^1)^

Item (%)	Australia	Indonesia
Dry matter	91.33	90.49
Gross energy (kcal/kg)	4,109	4,085
Crude protein	25.66	26.07
AEE	2.81	2.02
Ash	5.57	6.42
Carbohydrates		
Total starch	10.84	8.16
Insoluble dietary fiber	35.08	36.40
Soluble dietary fiber	1.38	2.19
Total dietary fiber	36.46	38.59
Glucose	3.21	1.59
Fructose	2.69	1.48
Maltose	2.57	2.50
Sucrose	1.39	2.81
Stachyose	ND	ND
Raffinose	0.31	0.23
Rest fraction^2)^	−1.51	0.14
Indispensable AA		
Arginine	1.01	1.09
Histidine	0.45	0.47
Isoleucine	0.83	0.91
Leucine	1.31	1.42
Lysine	1.17	1.31
Methionine	0.35	0.39
Phenylalanine	0.80	0.87
Threonine	0.80	0.87
Tryptophan	0.13	0.13
Valine	1.17	1.17
Dispensable AA		
Alanine	1.10	1.26
Aspartic acid	3.08	2.91
Cysteine	0.36	0.38
Glutamic acid	2.83	2.59
Glycine	0.94	1.04
Proline	1.32	1.38
Serine	0.74	0.77
Tyrosine	0.49	0.52
Minerals		
Calcium	0.13	0.12
Phosphorus	0.59	0.70
Magnesium	0.15	0.17
Potassium	1.84	1.45
Sodium	0.15	0.05
Sulfur	0.07	0.14
Cooper (mg/kg)	10.24	12.77
Iron (mg/kg)	107.08	85.33
Manganese (mg/kg)	51.03	62.02
Zinc (mg/kg)	77.13	99.44

AEE, acid hydrolyzed ether extract; ND, not detected; AA, amino acids.

1) Except for dry matter, all values were adjusted to 90% dry matter.

2) Rest fraction = calculated using the following equation: [Dry matter – (crude protein + AEE + ash + total dietary fiber + total starch + glucose + fructose + maltose + sucrose + stachyose + raffinose)].

**Table 3:** Analyzed nutrient composition of corn co-products, as-fed basis^1)^

Item (%)	Indonesia	Philippines	
	
	Corn gluten feed	Corn bran	Corn gluten meal
Dry matter	91.02	90.32	92.97
Gross energy (kcal/kg)	4,105	4,374	5,270
Crude protein	20.21	11.12	62.57
AEE	3.47	9.92	5.93
Ash	5.32	3.18	1.67
Carbohydrates			
Total starch	10.88	34.88	10.94
Insoluble dietary fiber	46.08	29.59	4.07
Soluble dietary fiber	1.19	1.20	1.16
Total dietary fiber	47.26	30.79	5.23
Rest fraction^2)^	2.86	0.11	3.66
Indispensable AA			
Arginine	0.96	0.69	2.11
Histidine	0.66	0.33	1.29
Isoleucine	0.63	0.38	2.70
Leucine	1.71	0.96	9.84
Lysine	0.73	0.53	1.16
Methionine	0.33	0.20	1.65
Phenylalanine	0.71	0.48	3.87
Threonine	0.71	0.42	2.10
Tryptophan	0.10	0.07	0.37
Valine	0.97	0.56	2.99
Dispensable AA			
Alanine	1.46	0.72	5.38
Aspartic acid	1.09	0.80	3.82
Cysteine	0.51	0.24	1.16
Glutamic acid	2.98	1.63	13.48
Glycine	0.90	0.54	1.85
Proline	1.80	0.78	5.76
Serine	0.72	0.44	2.94
Tyrosine	0.55	0.30	3.15
Minerals			
Calcium	0.04	0.25	0.01
Phosphorus	1.02	0.75	0.23
Magnesium	0.46	0.25	0.01
Potassium	1.65	0.63	0.13
Sodium	0.09	0.01	0.01
Sulfur	0.08	0.01	ND
Cooper (mg/kg)	7.74	23.61	9.75
Iron (mg/kg)	157.89	126.95	48.77
Manganese (mg/kg)	25.49	14.24	24.96
Zinc (mg/kg)	70.83	53.31	17.85

AEE, acid hydrolyzed ether extract; AA, amino acids; ND, not detected.

1) Except for dry matter, all values were adjusted to 90% dry matter.

2) Rest fraction = calculated using the following equation: [Dry matter – (crude protein + AEE + ash + total dietary fiber + total starch)].

**Table 4:** Analyzed nutrient composition of wheat bran, as-fed basis^1)^

Item (%)	Australia				Indonesia	Philippines	Thailand			Vietnam
	
	Sample 1	Sample 2	Sample 3	CV			Sample 1	Sample 2	CV	
Dry matter	89.45	89.8	88.47	0.77	88.99	88.81	90.13	88.74	1.10	90.80
Gross energy (kcal/kg)	4,161	4,057	3,980	2.24	4,100	4,130	4,114	4,058	0.97	4,092
Crude protein	18.50	17.35	17.39	3.70	15.38	15.90	18.85	15.05	15.86	16.03
AEE	3.85	4.08	4.36	6.24	3.93	4.43	5.16	3.26	32.04	4.56
Ash	4.48	4.46	4.97	6.30	3.81	4.65	4.75	3.51	21.29	5.16
Carbohydrates										
Total starch	20.02	18.94	12.41	24.05	22.55	16.21	14.08	26.06	42.22	17.35
Insoluble dietary fiber	34.61	36.18	42.62	11.23	35.50	42.26	40.94	31.85	17.67	40.84
Soluble dietary fiber	2.92	1.70	2.75	26.75	2.43	2.43	1.30	2.33	40.30	2.28
Total dietary fiber	37.53	37.88	45.37	11.00	37.93	44.69	42.24	34.18	14.92	43.12
Glucose	0.08	0.40	1.15	100.92	0.39	0.23	0.36	0.35	0.89	0.36
Fructose	0.18	0.28	0.68	69.52	0.25	0.19	0.25	0.25	1.10	0.30
Maltose	1.95	2.06	1.46	17.44	2.30	1.26	1.65	3.25	46.18	1.32
Sucrose	2.69	2.36	0.45	66.04	2.07	2.16	2.73	1.91	25.01	2.16
Stachyose	ND	0.07	ND	173.21	ND	0.05	0.06	ND	141.42	0.06
Raffinose	1.26	1.19	0.61	34.94	0.57	1.11	1.49	0.98	28.84	1.33
Rest fraction^2)^	−0.54	0.92	1.14	-	0.81	−0.89	−1.62	1.20	-	−1.73
Indispensable AA										
Arginine	1.17	1.11	1.09	3.59	0.98	1.01	1.18	0.84	23.56	0.96
Histidine	0.47	0.45	0.48	3.08	0.39	0.42	0.48	0.37	19.13	0.40
Isoleucine	0.58	0.54	0.57	3.82	0.52	0.52	0.57	0.50	9.58	0.50
Leucine	1.08	0.99	0.98	5.30	0.91	0.93	1.03	0.89	10.01	0.90
Lysine	0.67	0.69	0.67	1.61	0.63	0.66	0.74	0.56	19.75	0.64
Methionine	0.26	0.25	0.23	5.59	0.23	0.22	0.26	0.24	4.56	0.23
Phenylalanine	0.72	0.66	0.65	5.85	0.60	0.62	0.68	0.60	8.93	0.58
Threonine	0.54	0.52	0.51	3.35	0.48	0.49	0.54	0.45	13.34	0.48
Tryptophan	0.17	0.15	0.19	12.52	0.15	0.19	0.19	0.17	6.76	0.14
Valine	0.83	0.77	0.79	3.37	0.71	0.73	0.82	0.66	15.27	0.71
Dispensable AA										
Alanine	0.78	0.78	0.77	0.77	0.72	0.74	0.83	0.62	20.53	0.72
Aspartic acid	1.19	1.13	1.14	2.59	1.01	1.03	1.21	0.92	18.93	1.03
Cysteine	0.38	0.35	0.38	4.53	0.31	0.31	0.36	0.33	5.05	0.32
Glutamic acid	3.64	3.08	2.87	12.52	2.72	2.78	3.03	3.13	2.49	2.63
Glycine	0.93	0.87	0.96	4.65	0.80	0.82	0.93	0.70	19.88	0.78
Proline	1.16	0.99	0.94	11.18	0.90	0.90	0.96	0.99	2.56	0.84
Serine	0.67	0.62	0.60	6.02	0.55	0.55	0.63	0.53	12.44	0.55
Tyrosine	0.45	0.43	0.45	2.57	0.38	0.40	0.44	0.38	11.13	0.36
Minerals										
Calcium	0.10	0.09	0.09	6.02	0.12	0.09	0.10	0.07	23.89	0.04
Phosphorus	1.00	1.02	1.10	5.13	0.77	1.03	1.20	0.67	40.05	0.80
Magnesium	0.36	0.38	0.40	4.55	0.29	0.37	0.41	0.21	44.63	0.34
Potassium	1.06	1.01	1.16	7.03	0.96	1.19	1.07	0.75	24.72	0.75
Sodium	0.01	0.01	0.01	0.78	0.03	0.01	0.01	0.01	1.10	0.01
Sulfur	0.02	0.01	0.02	35.04	0.03	0.02	0.01	0.02	48.11	ND
Cooper (mg/kg)	20.92	20.72	22.61	4.87	17.72	19.60	23.56	14.58	33.27	18.46
Iron (mg/kg)	127.34	151.04	151.59	9.66	103.45	143.81	144.18	99.75	25.76	201.85
Manganese (mg/kg)	147.92	136.67	155.63	6.50	108.43	139.67	138.14	96.43	25.15	82.18
Zinc (mg/kg)	72.31	91.53	87.67	12.13	63.58	77.07	96.42	45.27	51.05	32.38

CV, coefficient of variation; AEE, acid hydrolyzed ether extract; ND, not detected; AA, amino acids.

1) Except for dry matter, all values were adjusted to 90% dry matter.

2) Rest fraction = calculated using the following equation: [Dry matter – (crude protein + AEE + ash + total dietary fiber + total starch + glucose + fructose + maltose + sucrose + stachyose + raffinose)].

**Table 5:** Analyzed nutrient composition of wheat mill mix and wheat pollard, as-fed basis^1)^

Item (%)	Australia	Philippines					
	
	Mill mix	Pollard soft	Pollard hard			CV	Average

			Sample 1	Sample 2	Sample 3		
Dry matter	90.52	91.26	90.00	91.30	89.44	1.03	90.50
Gross energy (kcal/kg)	4,144	4,036	4,084	4,032	4,129	1.12	4,070
Crude protein	16.67	16.12	17.67	17.61	18.66	5.97	17.52
AEE	4.50	4.38	4.69	4.01	4.65	7.06	4.43
Ash	3.98	4.79	5.12	4.21	4.97	8.35	4.77
Carbohydrates							
Total starch	18.00	20.02	14.80	24.64	17.50	21.75	19.24
Insoluble dietary fiber	38.68	36.19	39.60	30.76	36.13	10.25	35.67
Soluble dietary fiber	1.39	2.37	1.70	1.38	2.14	23.28	1.90
Total dietary fiber	40.07	38.56	41.30	32.14	38.26	10.30	37.56
Glucose	0.26	0.30	0.29	0.30	0.31	2.13	0.30
Fructose	0.15	0.21	0.20	0.19	0.20	4.32	0.20
Maltose	2.68	2.50	1.81	3.42	2.57	25.63	2.58
Sucrose	2.98	3.17	2.33	2.28	2.21	18.02	2.50
Stachyose	ND	ND	0.17	0.79	0.07	140.17	0.26
Raffinose	1.21	1.39	1.38	1.40	1.51	4.13	1.42
Rest fraction^2)^	−0.51	−1.43	0.24	−0.98	0.10	-	−0.52
Indispensable AA							
Arginine	1.03	1.07	1.11	1.08	1.23	6.43	1.13
Histidine	0.42	0.43	0.45	0.44	0.49	5.27	0.45
Isoleucine	0.54	0.53	0.53	0.60	0.62	8.18	0.57
Leucine	0.94	0.96	0.98	1.05	1.10	6.45	1.02
Lysine	0.66	0.68	0.67	0.67	0.77	7.18	0.70
Methionine	0.24	0.23	0.24	0.27	0.27	8.86	0.25
Phenylalanine	0.63	0.62	0.65	0.71	0.72	7.13	0.68
Threonine	0.50	0.48	0.52	0.51	0.56	6.07	0.52
Tryptophan	0.15	0.21	0.18	0.20	0.18	6.57	0.19
Valine	0.73	0.76	0.76	0.81	0.88	6.83	0.80
Dispensable AA							
Alanine	0.73	0.74	0.77	0.77	0.86	6.95	0.79
Aspartic acid	1.10	1.07	1.16	1.13	1.23	5.64	1.15
Cysteine	0.36	0.34	0.36	0.37	0.38	5.26	0.36
Glutamic acid	2.87	2.81	2.91	3.67	3.35	12.49	3.18
Glycine	0.84	0.82	0.87	0.86	0.96	6.65	0.88
Proline	0.94	0.87	0.91	1.13	1.05	12.42	0.99
Serine	0.59	0.54	0.62	0.61	0.64	7.08	0.60
Tyrosine	0.40	0.39	0.42	0.44	0.45	5.76	0.43
Minerals							
Calcium	0.09	0.09	0.10	0.09	0.10	7.43	0.09
Phosphorus	0.78	1.14	1.15	0.96	1.17	9.04	1.11
Magnesium	0.36	0.45	0.51	0.42	0.50	8.49	0.47
Potassium	0.99	1.38	1.28	1.06	1.28	10.65	1.25
Sodium	0.01	0.01	0.01	0.01	0.01	1.51	0.01
Sulfur	0.05	0.03	0.02	0.00	0.00	119.71	0.01
Cooper (mg/kg)	11.31	16.08	16.40	13.37	16.64	9.73	15.62
Iron (mg/kg)	131.57	133.63	161.44	119.10	335.12	53.43	187.32
Manganese (mg/kg)	152.89	139.30	154.46	125.28	163.90	11.66	145.74
Zinc (mg/kg)	65.33	74.55	92.13	78.87	100.08	13.65	86.41

CV, coefficient of variation; AEE, acid hydrolyzed ether extract; ND, not detected; AA, amino acids.

1) Except for dry matter, all values were adjusted to 90% dry matter.

2) Rest fraction = calculated using the following equation: [Dry matter – (crude protein + AEE + ash + total dietary fiber + total starch + glucose + fructose + maltose + sucrose + stachyose + raffinose)].

**Table 6:** Comparison among countries and ingredients for wheat co-products, as-fed basis^1)^

Item (%)	Countries				Wheat co-products^3)^			

	Wheat bran^2)^							
	
	Australia	Thailand	SEM	p-value	Wheat bran	Wheat pollard	SEM	p-value
Dry matter	89.24	89.44	0.52	0.807	89.40	90.50	0.36	0.058
Gross energy (kcal/kg)	4,066	4,046	50.19	0.577	4,086	4,072	24.00	0.667
Crude protein	17.75	16.87	1.08	0.570	16.81	17.52	0.57	0.402
AEE	4.10	4.21	0.52	0.887	4.20	4.43	0.22	0.474
Ash	4.64	4.13	0.41	0.480	4.47	4.77	0.22	0.367
Carbohydrates								
Total starch	17.12	20.07	3.83	0.624	18.45	19.24	1.90	0.775
Insoluble dietary fiber	37.80	36.40	3.28	0.781	38.10	35.67	1.70	0.337
Soluble dietary fiber	2.46	1.82	0.44	0.380	2.27	1.84	0.24	0.229
Total dietary fiber	40.26	38.21	3.16	0.677	40.37	37.57	1.72	0.277
Glucose	0.54	0.36	0.29	0.677	0.42	0.30	0.11	0.493
Fructose	0.38	0.25	0.14	0.557	0.30	0.20	0.06	0.262
Maltose	1.82	2.43	0.51	0.480	1.91	2.58	0.28	0.128
Sucrose	1.83	1.66	0.72	0.610	2.07	2.55	0.29	0.226
Stachyose	0.02	0.03	0.03	0.870	0.03	0.26	0.09	0.093
Raffinose	1.02	1.11	0.27	0.804	1.07	1.42	0.12	0.066
Rest fraction^4)^	0.51	0.03	0.81	0.596	−0.09	−1.08	0.47	0.075
Indispensable AA								
Arginine	1.12	1.01	0.10	0.459	1.04	1.12	0.05	0.243
Histidine	0.47	0.43	0.03	0.445	0.43	0.45	0.02	0.422
Isoleucine	0.56	0.54	0.02	0.296	0.54	0.57	0.02	0.186
Leucine	1.02	0.94	0.04	0.194	0.96	1.02	0.03	0.183
Lysine	0.68	0.65	0.05	0.720	0.66	0.70	0.02	0.228
Methionine	0.25	0.24	0.01	0.573	0.24	0.25	0.01	0.256
Phenylalanine	0.68	0.63	0.02	0.122	0.64	0.68	0.02	0.241
Threonine	0.52	0.49	0.03	0.464	0.50	0.52	0.01	0.429
Tryptophan	0.17	0.18	0.01	0.591	0.17	0.20	0.01	0.024
Valine	0.80	0.74	0.04	0.376	0.75	0.80	0.03	0.195
Dispensable AA								
Alanine	0.78	0.73	0.06	0.557	0.75	0.79	0.03	0.297
Aspartic acid	1.15	1.06	0.08	0.485	1.08	1.15	0.04	0.273
Cysteine	0.37	0.35	0.01	0.239	0.34	0.36	0.01	0.240
Glutamic acid	3.20	3.08	0.21	0.723	2.99	3.19	0.15	0.366
Glycine	0.92	0.82	0.07	0.380	0.85	0.88	0.04	0.578
Proline	1.03	0.98	0.06	0.571	0.96	0.99	0.04	0.647
Serine	0.63	0.57	0.03	0.222	0.59	0.60	0.02	0.621
Tyrosine	0.44	0.41	0.02	0.272	0.41	0.43	0.01	0.514
Minerals								
Calcium	0.09	0.08	0.01	0.463	0.09	0.08	0.01	0.725
Phosphorus	1.04	0.94	0.14	0.638	0.95	1.11	0.07	0.145
Magnesium	0.38	0.31	0.05	0.425	0.35	0.47	0.03	0.007
Potassium	1.08	0.92	0.10	0.382	0.99	1.25	0.07	0.025
Sodium	0.01	0.01	0.00	0.998	0.01	0.01	0.00	0.506
Sulfur	0.01	0.02	0.01	0.591	0.00	0.01	0.00	0.119
Cooper (mg/kg)	21.42	19.09	2.68	0.599	19.77	15.62	1.10	0.024
Iron (mg/kg)	143.32	121.97	13.80	0.354	140.38	187.32	26.44	0.238
Manganese (mg/kg)	146.74	119.16	12.63	0.241	125.63	145.74	10.41	0.202
Zinc (mg/kg)	83.84	70.85	14.51	0.572	70.78	86.41	0.23	0.231

SEM, standard error of the means; AEE, acid hydrolyzed ether extract; AA, amino acids.

1) Except for dry matter, all values were adjusted to 90% dry matter.

2) For wheat bran from Australia there were 3 observations and for wheat bran from Thailand there were 2 observations.

3) There were 8 observations for wheat bran and 4 observations for wheat pollard.

4) Rest fraction = calculated using the following equation: [Dry matter – (crude protein + AEE + ash + total dietary fiber + total starch + glucose + fructose + maltose + sucrose + stachyose + raffinose)].
